# Source of Purchased Medications and Its Impact on Medication Mistakes and Hospitalizations: The NHATS 2017

**DOI:** 10.1093/geroni/igaa057.2875

**Published:** 2020-12-16

**Authors:** Martha Coates, Janeway Granche, Rose Ann DiMaria-Ghalili

**Affiliations:** Drexel University, Philadelphia, Pennsylvania, United States

## Abstract

Older adults self-administer prescribed medication regimens to treat chronic diseases which can lead to mismanagement, medication related harm and hospitalizations. We examined the extent to which source of purchased medications influenced the occurrence of self-reported medication mistakes and hospitalizations in community-dwelling participants who managed medications independently (N= 3899). The majority (65%) picked-up medications, 18% had medications delivered, and 17% used both (picked-up and delivery). Compared to those picking up their medications, those using delivery only were less likely to have a hospital stay (OR=0.691 [95% CI 0.507-0.943]) and no difference in odds of medication mistakes (OR=1.051 [95% CI 0.764-1.445]), while those using both methods were more likely to report hospital stays (OR=1.429 [95% CI 1.106-1.846]) and medication mistakes (OR = 1.576[95% CI 1.078-2.304]). Older adults who picked-up medications from a local pharmacy and had medications delivered were more likely to report medication mistakes and hospitalizations.

